# Purification and characterization of the receptor‐binding domain of SARS‐CoV‐2 spike protein from *Escherichia coli*

**DOI:** 10.1002/elsc.202000106

**Published:** 2021-05-07

**Authors:** Yunxia He, Jinming Qi, Lucheng Xiao, Lijuan Shen, Weili Yu, Tao Hu

**Affiliations:** ^1^ State Key Laboratory of Biochemical Engineering Institute of Process Engineering Chinese Academy of Sciences Beijing P. R. China; ^2^ University of Chinese Academy of Sciences Beijing P. R. China

**Keywords:** COVID‐19, purification, RBD, SARS‐CoV‐2, spike protein

## Abstract

SARS‐CoV‐2 is responsible for a disruptive worldwide viral pandemic, and renders a severe respiratory disease known as COVID‐19. Spike protein of SARS‐CoV‐2 mediates viral entry into host cells by binding ACE2 through the receptor‐binding domain (RBD). RBD is an important target for development of virus inhibitors, neutralizing antibodies, and vaccines. RBD expressed in mammalian cells suffers from low expression yield and high cost. *E. coli* is a popular host for protein expression, which has the advantage of easy scalability with low cost. However, RBD expressed by *E. coli* (RBD‐1) lacks the glycosylation, and its antigenic epitopes may not be sufficiently exposed. In the present study, RBD‐1 was expressed by *E. coli* and purified by a Ni Sepharose Fast Flow column. RBD‐1 was structurally characterized and compared with RBD expressed by the HEK293 cells (RBD‐2). The secondary structure and tertiary structure of RBD‐1 were largely maintained without glycosylation. In particular, the major β‐sheet content of RBD‐1 was almost unaltered. RBD‐1 could strongly bind ACE2 with a dissociation constant (K_D_) of 2.98 × 10^–8^ M. Thus, RBD‐1 was expected to apply in the vaccine development, screening drugs and virus test kit.

**Abbreviations**
ACE2: angiotensin‐converting enzyme 2
ANS: 1‐anilino‐8‐naphthalene sulfonic acid
CD: circular dichroism
EF: extrinsic fluorescence
FT‐IR: Fourier‐transform infrared spectroscopy
K_D_: dissociation constant
RBD: receptor‐binding domain
RBD‐1: RBD derived from *E. coli*
RBD‐2: RBD derived from the HEK293 cells
SEC: size exclusion chromatography
SPR: surface plasmon resonance

## 1 INTRODUCTION

SARS‐CoV‐2 belongs to the β‐coronavirus genus, which is a member of the SARS‐related coronavirus [1, 2]. SARS‐CoV‐2 is responsible for a disruptive worldwide viral pandemic and a severe respiratory disease known as COVID‐19 [3, 4]. COVID‐19 is now widespread around the globe and spreads readily with an exponential increase in recent days [5]. COVID‐19 prominently affects the respiratory tract, with the initial symptoms of common cold, fever, dry cough, fatigue, nasal congestion, sore throat, and diarrhea to severe pneumonia [6, 7].

There is an urgent quest to develop effective therapeutics and preventive vaccines against SARS‐CoV‐2 [8, 9]. SARS‐CoV‐2 contains four structural proteins, including spike (S), envelope, membrane and nucleocapsid proteins [10, 11]. S protein plays the most important roles in viral attachment, fusion, and entry. It serves as a target for development of antibodies, entry inhibitors and vaccines [12, 13]. In addition, it can bind to a host receptor, angiotensin‐converting enzyme 2 (ACE2) through the receptor‐binding domain (RBD) [14]. Thus, RBD of SARS‐CoV‐2 S protein is an appealing antigen for vaccine development, which elicits most neutralizing antibodies during SARS‐CoV‐2 infection [15]. Moreover, an advantage of the RBD‐based vaccine is its ability to minimize the host immunopotentiation [16].

RBD contains 220 amino acid residues with nine cysteine residues and two N*‐*glycosylation sites [17]. The apparent molecular mass of RBD was determined to be ∼34 kDa, whereas that of the RBD amino acid sequence alone was ∼27 kDa [17]. N‐glycosylation and O‐glycosylation were both observed by analysis of RBD [18]. The glycan moieties have a relevant role in the in vivo protein folding process, stability, and immunogenicity of RBD [19]. RBD has a central twisted antiparallel β‐sheet formed by five strands decorated with secondary structure elements and loops [20].

RBD has been expressed in the eukaryotic cell expression systems, including baculovirus‐insect cells, yeast cells, and mammalian cells (e.g. HEK293 cells and CHO cells) [21, 22]. However, the low expression yield and high cost of RBD could not meet the need of the therapeutics and vaccine development [23]. Bacterial expression systems for heterologous protein expression have the advantages of easy use, low cost, short generation times, and scalability [24]. Particularly, *E. coli* is one of the most popular bacterial hosts for heterologous protein expression [25].

Some studies suggest that RBD of SARS‐CoV S protein expressed by *E. coli* could provide protective immunity [26, 27]. Moreover, RBD (N318‐V510) of SARS‐CoV‐2 S protein was expressed by *E. coli* and has been used as a cost‐effective antigen for worldwide serological testing [28]. However, RBD of SARS‐CoV‐2 expressed in *E. coli* (RBD‐1) lacks the disulfide bond formation and glycosylation. Thus, the expression and protein folding of RBD‐1 are different to the one expressed in HEK293 cells (RBD‐2). Because *E. coli* is cost‐efficient for expression of RBD, it is of interest to investigate the structure and ACE2‐binding affinity of RBD‐1.

**PRACTICAL APPLICATION**

The receptor‐binding domain (RBD) of SARS‐CoV‐2 spike protein is a vital target in mediating the entry of the virus into the host cells. RBD was mainly expressed by the eukaryotic cell expression systems, which suffered from the high cost and low expression level of RBD derived from the eukaryotic cell limit its practical application. In contrast, RBD expressed by *E. coli* (RBD‐1) has the advantage of easy scalability with low cost. In the present study, RBD‐1 was expressed, purified, and structurally characterized. The secondary and tertiary structure of RBD‐1 was largely maintained. The binding affinity of RBD‐1 to angiotensin‐converting enzyme 2 (ACE2) was measured with a dissociation constant (K_D_) of 2.98 × 10^–8^ M. Our study is of practical significance for SARS‐CoV‐2 vaccine development, drug screening and virus test kit.

In the present study, RBD‐1 was expressed by *E. coli*, renatured, and purified by a Ni Sepharose Fast Flow column. The structure of RBD‐1 was characterized and its binding affinity to ACE2 was measured. The properties of RBD‐1 were compared with RBD‐2 to evaluate its effectiveness for the vaccine design, screening drugs, therapeutics, and virus test kit.

## 2 MATERIALS AND METHODS

### 2.1 Materials

Human angiotensin‐converting enzyme 2 (ACE2) was purchased from Sino Biological (Beijing, China). *N*‐hydroxysuccinimide (NHS), 1‐anilino‐8‐naphthalene sulfonic acid (ANS) and 1‐(3‐dimethylaminopropyl)‐3‐ethylcarbodiimide hydrochloride (EDC) were purchased from Sigma‐Aldrich (USA).

### 2.2 Expression of RBD in HEK293 cells

Recombinant RBD of SARS‐CoV‐2 S protein (RBD‐2) was expressed by the HEK293 cells [17] and gifted from Academy of Military Medical Sciences (Beijing, China).

### 2.3 Construction of RBD expression vector

The coding DNA sequence of RBD of SARS‐CoV‐2 S protein (amino acid 330–583 of S protein) was from strain Wuhan‐Hu‐1 (Genbank Acc. No. 045512.2). The sequence was amplified by PCR and cloned into the *p*ET28a bacterial expression vector with a C‐terminal His‐tag. Correct DNA sequences were confirmed by restriction digestion and DNA sequence analysis. The resulting plasmid was transformed into *E. coli* host BL‐21 (DE3) cells. The expression strain was screened out by streaking the plate and cultured in 5 mL LB medium.

### 2.4 Expression of RBD‐1 in *E. coli*

The expression strain was incubated in the LB medium (50 mL) for overnight and then in the LB medium (1 L) containing 0.1% kanamycin. The resultant culture was incubated with vigorous aeration at 37°C until the strain density was up to the absorbance range of 0.6–0.8 at 600 nm. IPTG was added into the culture at a final concentration of 0.5 mM and then incubated at 37°C for 3 h. Then, the cells were harvested by centrifugation at 4000 rpm for 35 min. The pellet was resuspended in 50 mM Tris‐HCl buffer (pH 8.0) and sonicated in an ice bath. Finally, the inclusion bodies containing RBD were collected by centrifugation at 8000 rpm for 30 min.

### 2.5 Refolding and purification of RBD‐1

The inclusion bodies were washed with 50 mM Tris‐HCl buffer (pH 8.0) containing 1 M NaCl and 2 M urea. Then, they were solubilized by 50 mM Tris‐HCl buffer (pH 8.0) containing 1 mM EDTA, 15 mM DTT, and 6 M GuHCl. RBD in the inclusion bodies was refolded by dropwise dilution of a refolding buffer with constant stirring overnight at 4°C. The refolding buffer was 20 mM sodium phosphate buffer (PB buffer, pH 8.0) containing 0.18 mM EDTA, 0.5 M l‐arginine, 1.8 mM GSH, 0.9 mM GSSG, and 2 M urea for overnight at 4°C.

RBD was purified from the refolding solution by a Ni Sepharose Fast Flow column (0.5 cm × 5.0 cm, GE Healthcare, USA). The column was equilibrated with five column volumes (CVs) of PB buffer containing 1 M urea and 20 mM imidazole (buffer A, pH 8.0) and eluted by a gradient elution with 10 CVs of 0–0.8 M imidazole in buffer A. The peak corresponding to RBD was fractionated.

### 2.6 SDS‐PAGE analysis

RBD‐1 and RBD‐2 were both analyzed by SDS‐PAGE, using an 12% SDS‐polyacrylamide gel under a reducing (5% β‐mercaptoethanol, v/v) condition. The molecular weights of RBD‐1 and RBD‐2 were estimated with suitable markers. The gel was stained with Coomassie blue R‐250.

### 2.7 Size exclusion chromatography analysis

Size exclusion chromatography (SEC) analysis of RBD‐1 and RBD‐2 was carried out on an analytical Superdex 200 column (1 cm × 30 cm, GE Healthcare, USA). The column was equilibrated and eluted with PB buffer (pH 7.4) at a constant flow rate of 0.5 mL/min. RBD‐1 and RBD‐2 were both injected at a volume of 100 μL and a protein concentration of 1 mg/mL. The effluent was detected at 280 nm.

### 2.8 Circular dichroism spectroscopy

The secondary structures of RBD‐1 and RBD‐2 were measured by the far‐UV circular dichroism (CD) spectroscopy, using a Jasco‐810 spectropolarimeter (Jasco, Japan). The spectra were recorded between 260 and 190 nm. The two proteins were both at a protein concentration of 0.3 mg/mL in PB buffer (pH 7.4). The spectra represented an average of three individual scans and were corrected for absorbance caused by the buffer. A quartz cuvette with a 0.1 cm path length was used for the measurement. The secondary structure data of the two proteins were obtained by the structure fitting software that has been built‐into the JASCO J‐810 spectropolarimeter.

### 2.9 Fluorescence spectroscopy

Intrinsic fluorescence measurements of RBD‐1 and RBD‐2 were carried out on a Hitachi F‐4500 fluorescence spectropolarimeter (Hitachi, Japan) at room temperature. The intrinsic emission spectra were recorded from 300 to 400 nm and excited at 280 nm. A quartz cuvette with a 1.0 cm path length was used for the measurement. Excitation slit width was 5 nm and emission slit width was 10 nm. RBD‐1 and RBD‐2 were both at a protein concentration of 0.1 mg/mL in PB buffer (pH 7.4).

RBD‐1 and RBD‐2 were both determined by extrinsic fluorescence measurement, using ANS as the fluorescence probe. RBD‐1 and RBD‐2 were mixed with 10‐fold molar ANS at a protein concentration of 0.1 mg/mL in PB buffer (pH 7.4), respectively. The emission spectra were recorded from 400 nm to 650 nm at a scan rate of 120 nm/min and excited at 350 nm. A quartz cuvette with a 1.0 cm path length was used for the measurement. Excitation slit width was 5 nm and emission slit width was 10 nm.

### 2.10 Fourier‐transform infrared spectroscopy

RBD‐1 and RBD‐2 were both analyzed by Fourier‐transform infrared spectroscopy (FT‐IR), using a Nicolet iS50 FT‐IR spectrometer (Thermo Fisher, USA). The two proteins were both lyophilized and mixed with KBr, followed by pressing into a thin tablet. The FT‐IR spectra were scanned from 4000 to 400 cm^–1^, and the interferograms were presented as transmittance.

### 2.11 Interaction of RBD‐1 with ACE2

The interaction between RBD‐1 and ACE2 was determined by surface plasmon resonance (SPR), using a BIAcore T100 instrument (GE Healthcare, Boston, USA). ACE2 was immobilized on the surface of a CM5 sensor chip, using the cross‐linker EDC and NHS. RBD‐1 and RBD‐2 were measured at a flow rate of 10 μL/min with an association phase of 1 min after injection, followed by dissociation for 3 min. RBD‐1 and RBD‐2 were diluted by the filtered and degassed PBST running buffer (PBS buffer containing 0.5% Tween‐20, pH 7.4). RBD‐1 and RBD‐2 were both injected at a concentration range of 12.5‐200 nM. Dose‐response data were collected in the single cycle format [29]. The data were automatically fitted to the 1:1 (Langmuir) binding model for both kinetics and steady‐state affinity.

## 3 RESULTS

### 3.1 Expression of RBD‐1

**FIGURE 1:**
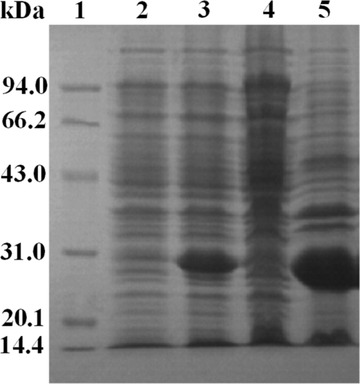
SDS‐PAGE analysis of the cell culture. Lane 1, standard marker; Lane 2, the culture without IPTG induction; Lane 3, the culture with IPTG induction; Lane 4, the supernatant; Lane 5, the inclusion bodies

RBD‐1 was induced and expressed in *E. coli* BL‐21 cells. The cells were harvested and sonicated to obtain the cell‐free extract for SDS‐PAGE analysis. As shown in Figure 1, a major electrophoresis band corresponding to ∼27 kDa was observed in the culture with IPTG induction (Lane 3), which was absent in the culture without IPTG induction (Lane 2). Thus, RBD‐1 was expressed in *E. coli* upon induction with IPTG. Minor RBD‐1 was also detected in the supernatant (Lane 4). However, RBD‐1 was predominantly in the form of insoluble inclusion bodies (Lane 5). The inclusion bodies were solubilized and renatured for purification.

### 3.2 Purification of RBD‐1

**FIGURE 2:**
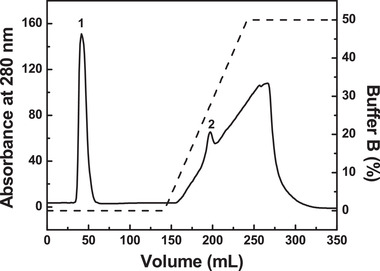
Purification of RBD‐1 by a Ni Sepharose Fast Flow column. The column (0.5 cm × 5.0 cm) was equilibrated with five CVs of PB buffer containing 1 M urea and 20 mM imidazole (buffer A, pH 8.0) and eluted by a gradient elution with 10 CVs of 0–0.8 M imidazole in buffer A

The inclusion bodies containing RBD‐1 were subsequently dissolved, renatured and purified by a Ni Sepharose Fast Flow column (0.5 cm × 5.0 cm). As shown in Figure 2, a major single peak (peak 1) was observed upon elution with PB buffer containing 1 M urea and 20 mM imidazole (pH 8.0), which could remove the most unbound protein. A separated minor peak (peak 2) was observed upon gradient salt elution, corresponding to the bound RBD‐1. The fractions corresponding to peak 2 were pooled and concentrated. The recovery rate of RBD‐1 after the purification process was 65.2%.

### 3.3 SDS‐PAGE analysis

**FIGURE 3:**
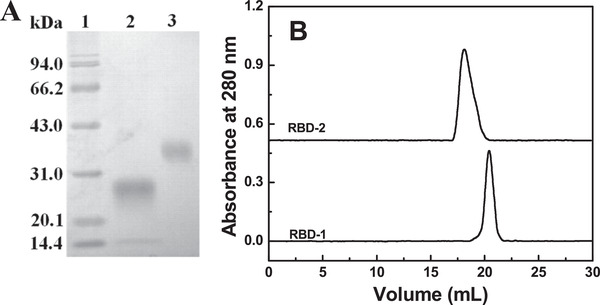
Characterization of RBD‐1. The two proteins were characterized by SDS‐PAGE (A). Lane 1: Marker; Lane 2: RBD‐1; Lane 3: RBD‐2. The two proteins were analyzed by a Superdex 200 column (1 cm × 30 cm) at room temperature (B)

As shown in Figure 3A, RBD‐1 (Lane 2) exhibited a single electrophoresis band, corresponding to an apparent Mw of ∼27.0 kDa. RBD‐2 (Lane 3) displayed a single electrophoresis band with a slower migration rate, corresponding to an apparent Mw of ∼35 kDa. This indicated the high purity of RBD‐1 and RBD‐2. The larger Mw of RBD‐2 was due to glycosylation of the protein expressed by the HEK‐293 cells.

### 3.4 Size exclusion chromatography analysis

RBD‐1 and RBD‐2 were both analyzed by an analytical Superdex 200 column (1 cm×30 cm), based on size exclusion chromatography. As shown in Figure 3B, RBD‐1 was eluted as a single and narrow elution peak at 20.4 mL. In contrast, RBD‐2 was eluted as a single and broad elution peak at 18.0 mL. As compared with RBD‐1, the left‐shifted peak of RBD‐2 was due to the glycosylation of RBD.

### 3.5 Fluorescence spectroscopy

**FIGURE 4:**
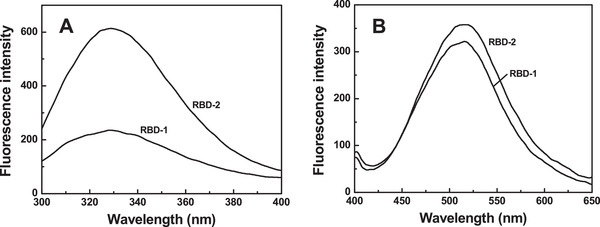
Fluorescence spectroscopy analysis of RBD‐1. The intrinsic fluorescence emission spectra (A) were recorded from 300 to 400 nm. The extrinsic fluorescence emission spectra (B) were recorded from 400 to 650 nm

Fluorescence spectroscopy was used to monitor the tertiary structure of RBD‐1 and RBD‐2. As shown in Figure 4A, RBD‐2 exhibited a single broad peak with a maximum emission fluorescence intensity at 328 nm when the excitation wavelength was at 280 nm. In contrast, the intrinsic fluorescence intensity of RBD‐1 was much lower than that of RBD‐2. Presumably, the disulfide bonds may be formed for the Cys‐abundant RBD‐1 and rendered the quenching effect on the fluorescence intensity of RBD‐1 [29]. The maximum fluorescence intensities of RBD‐2 were both at 328 nm. This indicated that the tertiary structure of RBD‐1 was comparable to that of RBD‐2.

RBD‐1 and RBD‐2 were mixed with a fluorescent probe (ANS), respectively. The hydrophobicity of RBD‐1 and RBD‐2 was thus determined by measuring the extrinsic fluorescence (EF) intensities of the mixtures. As shown in Figure 4B, RBD‐2 showed a single and broad peak with a maximum EF intensity at 516 nm. In contrast, RBD‐1 displayed a slightly lower peak with a maximum EF intensity at 518 nm. This suggested that glycosylation expressed in the HEK‐293 cells made the hydrophobic residues of RBD slightly less exposed to the surrounding environment. This lowered the EF intensity and rendered that the intensity peak of RBD‐1 was red‐shifted.

### 3.6 Circular dichroism spectroscopy

**FIGURE 5:**
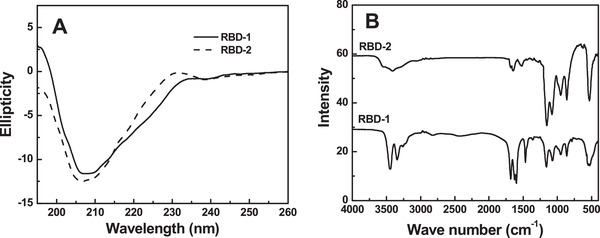
Structural characterization of RBD‐1. The CD spectra (A) were recorded from 260 to 190 nm. FT‐IR spectra (B) was obtained from 4000 to 500 cm^–1^

The secondary structures of RBD‐1 and RBD‐2 were measured by far‐UV CD spectroscopy. As shown in Figure 5A, the CD spectra of RBD‐1 and RBD‐2 both displayed a single negative maximum ellipticity around 208 nm, a characteristic of β‐sheet structure. The negative maximum ellipticity of RBD‐2 around 208 nm was slightly larger than that of RBD‐1. In particular, the β‐sheet percentages of RBD‐1 and RBD‐2 were 73.7% and 74.7%, respectively. The random and α‐helix contents of RBD‐1 were 20.7% and 4.6%, respectively. The random and α‐helix contents of RBD‐2 were 22.9% and 2.0%, respectively. This indicated that the secondary structure of RBD‐2 was slightly less dense than that of RBD‐1.

### 3.7 FT‐IR spectroscopy

FT‐IR spectroscopy was used to structurally analyze RBD‐1 and RBD‐2. As shown in Figure 5B, the spectra of the two proteins both displayed the characteristic peaks at 949 cm^–1^ (O—H wagging), 1159 cm^–1^ (C—O stretching), 1467 cm^–1^ (C—O vibration) and 1699 cm^–1^ (C=O stretching). RBD‐1 showed two sharp peaks at 3443 and 3346 cm^–1^, which assigned to N—H stretching in primary aliphatic amines. As compared with RBD‐1, the peak at 3443 cm^–1^ was shifted to 3552 cm^–1^ (O‐H stretching) for RBD‐2. Moreover, the signal of RBD‐2 at 949 cm^–1^ was stronger than that of RBD‐1. This indicated that the hydroxyl content of RBD‐1 was lower than that of RBD‐2, possibly related to the absence of glycosylation in RBD‐1.

### 3.8 SPR analysis of RBD‐1 and RBD‐2

**FIGURE 6:**
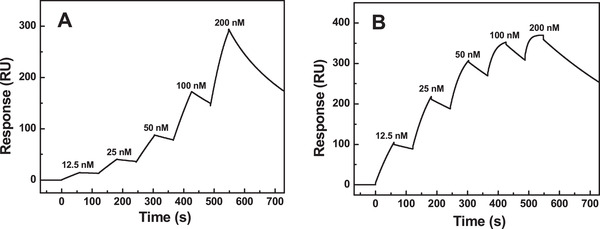
SPR analysis of the interaction between RBD and ACE2. RBD‐1 (A) and RBD‐2 (B) were measured at a flow rate of 10 μL/min with an association phase of 1 min after injection, followed by dissociation for 3 min. Dose‐response data were collected in the single cycle format

The kinetics for the binding of two proteins to ACE2 was determined by the SPR analysis. The kinetic curves were shown in Figure 6. The association rate (k_a_), dissociation rate (k_d_), and dissociation constant (K_D_, k_d_/k_a_) were calculated from the kinetic curves. ACE2‐RBD‐2 showed a k_a_ of 4.51 × 10^5^ M^–1^ s^–1^, a k_d_ of 1.92 × 10^–3^ s^–1^, and a K_D_ of 4.25 × 10^–9^ M (Figure 6B). In contrast, ACE2‐RBD‐1 showed a k_a_ of 2.81 × 10^5^ M^–1^ s^–1^, a k_d_ of 8.38 × 10^–3^ s^–1^, and a K_D_ of 2.98 × 10^–8^ M (Figure 6A). Thus, the kinetic result suggested that RBD‐1 could strongly bind with ACE2.

## 4 DISCUSSION

At present, several SARS‐CoV‐2 vaccine candidates have been developed, using RBD of SARS‐CoV‐2 S protein as the antigen. The RBD antigen was mainly expressed by the eukaryotic cell expression systems, such as mammalian cells, insect cells, and yeast cells. However, RBD‐2 derived from the eukaryotic cells suffered from high cost and low expression level. As compared with RBD‐2, RBD‐1 expressed by *E. coli* was greatly scalable at a low cost. In the present study, the product yield of RBD‐1 was 13.3 mg/L by flask culture. In contrast, the product yield of RBD‐2 in mammalian cells (HEK‐293T) was 5 mg/L by cell culture [30]. However, the effectiveness of RBD‐1 derived from *E. coli* was necessary to be evaluated.

In the present study, RBD‐1 was expressed by *E. coli* in the form of inclusion bodies. The inclusion bodies were dissolved in 6 M guanidine hydrochloride and renatured in the presence of 0.5 M l‐arginine. The renatured RBD was purified by a Ni Sepharose Fast Flow column. RBD was thus obtained by one‐step affinity purification process with high purity and high purification yield. As compared with RBD expressed by the HEK293 cells (RBD‐2), RBD expressed by *E. coli* (RBD‐1) lacks the glycosylation. The absence of glycosylation correlated with the decreased size of RBD‐1, which may shorten the serum duration of RBD. If RBD was formulated and used as nanoparticle vaccines, the size effect of RBD could be neglected.

The structure of RBD‐1 was investigated by CD, fluorescence and FT‐IR. CD suggested that the major β‐sheet content of RBD‐1 was almost unaltered. Fluorescence spectroscopy suggested that the tertiary structure of RBD‐1 was slightly changed. FT‐IR spectroscopy revealed that RBD‐1 lacked the glycosylation with a slight structural alteration. SPR analysis suggested that RBD‐1 could strongly bind ACE2 with a K_D_ of 2.98 × 10^–8^ M. Thus, our study was of practical significance to ensure the effectiveness of RBD for clinical application.

In summary, RBD‐1 was successfully expressed in *E. coli* and purified by Ni affinity chromatography. RBD‐1 was structurally characterized and compared with RBD expressed by the HEK293 cells (RBD‐2). The secondary structure and tertiary structure of RBD‐1 were largely maintained. Moreover, RBD‐1 could strongly bind ACE2 with a K_D_ of 2.98 × 10^–8^ M. Thus, RBD‐1 was expected to apply in the vaccine design, screening drugs and virus test kit.

## CONFLICT OF INTEREST

The authors have declared no conflict of interest.

## Data Availability

The data that support the findings of this study are openly available in *Engineering in Life Sciences*, reference number (elsc. 202000106. R2).
